# Gelsolin suppresses gastric cancer metastasis through inhibition of PKR-p38 signaling

**DOI:** 10.18632/oncotarget.10557

**Published:** 2016-07-13

**Authors:** Xiangliang Yuan, Weiwei Wang, Junhua Li, Peiming Zheng, Ping Dong, Lei Chen, Yunlan Zhou, Guohua Xie, Dakang Xu, Yingbin Liu, Lisong Shen

**Affiliations:** ^1^ Department of Clinical Laboratory, Xinhua Hospital, Shanghai Jiao Tong University School of Medicine, Shanghai 200092, China; ^2^ Department of Surgery, Xinhua Hospital, Shanghai Jiao Tong University School of Medicine, Shanghai 200092, China; ^3^ MIMR-PHI Institute of Medical Research, Monash University, Clayton, Victoria 3168, Australia; ^4^ Institute of Ageing Research, Hangzhou Normal University School of Medicine, Hangzhou, Zhejiang 311121, China

**Keywords:** gelsolin, gastric cancer, metastasis, PKR, p38MAPK protein kinase

## Abstract

The biological function of gelsolin in gastric cancer and its mechanism remained undefined. Here, we demonstrated that gelsolin was down-regulated in human gastric cancer tissues, and lower tumorous gelsolin significantly correlated with gastric cancer metastasis. Functionally, gelsolin suppressed the migration of gastric cancer cells in *vitro* and inhibited lung metastasis in *vivo*. In mechanism, gelsolin decreased epithelial–mesenchymal transition (EMT) inducing cytoskeleton remolding through inhibition of p38 signaling to suppress the migration of gastric cancer cell. Moreover, gelsolin bound to and decreased the phosphorylation of PKR, and then inhibited p38 signaling pathway. Finally, similar to the gastric cancer cell lines, PKR-p38 signaling pathway proteins tend to be activated and correlated with low expression of gelsolin in clinical gastric cancer tissues. Altogether, these results highlight the importance of gelsolin in suppression of gastric cancer metastasis through inhibition of PKR-p38 signaling pathway.

## INTRODUCTION

Gastric cancer (GC) is the second leading cause of cancer-related deaths worldwide [1], the death rates from gastric cancer are high especially in Asian countries [2, 3]. Therefore, the understanding of the detailed mechanism involved in progression of gastric cancer would be helpful to improve treatment. So far, less is known about the molecular mechanism that is responsible for the conversion of a noninvasive gastric neoplasm to one with an invasive phenotype. In solid tumor, the spread of tumor cells is critically dependent on the integration of migratory and invasive signals that involve the cytoskeleton and extracellular matrix (ECM) remodeling [4, 5].

Gelsolin is an actin-binding protein that serves to sever and cap actin filaments and that regulates cytoskeletal turnover [6]. Published data indicated that gelsolin was downregulated in several solid tumors [7–11]. Our previous data also showed that the gelsolin were decreased in bladder cancer and regulated by ATF3 [12, 13]. Several *in vivo* and *in vitro* studies have indicated that gelsolin is crucial for the migration and invasion of tumor cells [14–16]. However, its migratory and invasive activities in epithelial cancers are unclear and may involve a combination of mechanisms that include migration and interactions with signaling proteins. Moreover, new insights have been discovered that gelsolin participates in the coordinated regulation of several signal transduction pathways and functional upstream in a variety of signaling processes [6]. Recently, gelsolin has been shown to be involved in signal transduction, acting as a potential binding partner to HPV-16 E7 [17]. The decreased expression of gelsolin has been reported in gastric cancer [10], however, the role and detailed mechanism of gelsolin in the pathogenesis of gastric cancer has been under explored. Thus, it is essential to identify the function of gelsolin in gastric cancer.

This study aims to address the gap in knowledge between the role of gelsolin and the signaling transduction process during the metastasis of gastric cancer cells. We investigated the influence of gelsolin on gastric cancer progression as well as the mechanism that underlie its activity.

## RESULTS

### Gelsolin is down-regulated in gastric cancer and associated with advanced clinical stage and metastasis status

**Figure 1:**
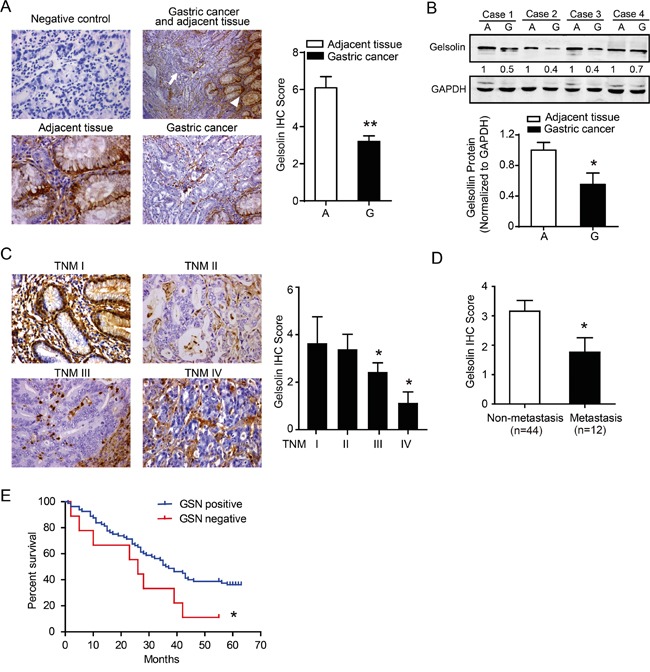
Downregulated gelsolin negatively correlated with tumor stage in gastric cancer **A.** Immunohistochemistry (IHC) staining of gelsolin in human paraffin-embedded gastric cancer and adjacent tissues. IgG_1_ was used for the negative control of gelsolin (upper left, 400x). Representative IHC image of gelsolin in gastric cancer (arrow) and adjacent tissues (arrowhead) (upper right, 200x). Magnified image showed the different expression pattern of gelsolin in adjacent tissues (bottom left, 400x) and gastric cancer (bottom right, 400x). Statistical analyses of the average score of gelsolin staining between GC tissues (G) and corresponding adjacent tissues in 20 cases (A). (**, *P*<0.01). **B.**, immunoblotting (IB) analysis of gelsolin expression in 4 cases of human gastric cancer tissues (G) and matched adjacent tissues (A). Quantification of IB analyses for gelsolin in 20 cases of matched human tissues was conducted (*, *P*<0.05).**C.**, Representative IHC images of gelsolin expression in different stages of gastric cancer tissues (TNM I, II, III, IV) (400x).(*, *P*<0.05 compared to TNM-I). **D.**, Statistical analyses of average score of gelsolin according to the metastasis status (*, *P*<0.05). **E.**, Kaplan–Meier survival curve analyses of 56 patients with gastric cancer determined by IHC scoring of gelsolin as negative (score=0) versus positive (score >0). (*, *P*<0.05).

To identify the expression level of gelsolin, a human gastric cancer tissue microarray (TMA) was performed by immunohistochemistry (IHC). We found that gelsolin expression was suppressed in gastric cancer compared to their corresponding non-tumorous tissues (Figure 1A). Western blot analysis confirmed the down-regulated gelsolin in gastric cancer tissues (Figure 1B). We also found that gastric cancer with advanced stages (stage III and IV) had a lower gelsolin expression than gastric cancer with early stages (stage I and II; Figure 1C). Interestingly, when all gastric cancer samples were stratified on the basis of the status of metastasis, we found that gelsolin expression was further significantly down-regulated in gastric cancer that had metastasis, when compared with those that did not have (*P*<0.05, Figure 1D). We then evaluated the association between gelsolin expression and survival in our enrolled patients with gastric cancer. Negative expression of gelsolin was associated with a poor outcome following surgical resection of gastric cancer in our clinical cohort (Figure 1F). Our data also showed that DNA methylation and histone deacetylation might account for the down-regulation of gelsolin in gastric cancer (Supplementary Figure S1).Collectively, the above findings suggest that loss of gelsolin is a frequent event in gastric cancer and may play a critical role in gastric cancer progression.

### Gelsolin inhibits the migration of gastric cancer cells

**Figure 2:**
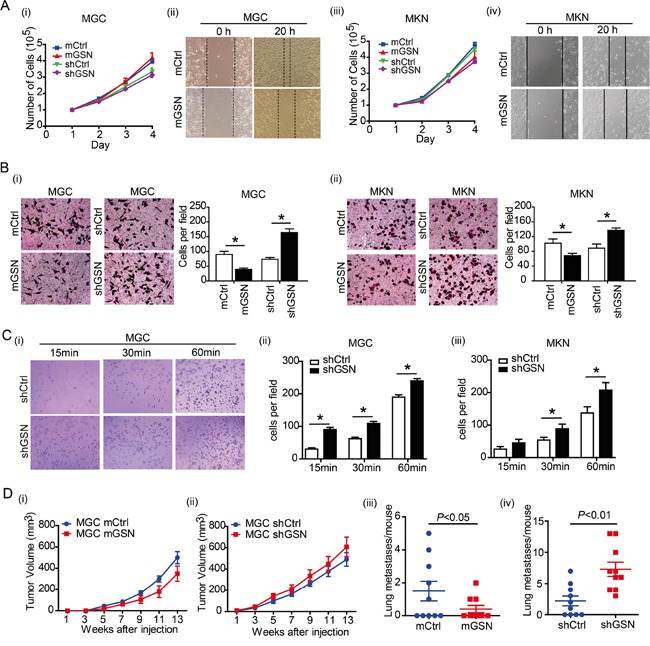
Gelsolin suppress the migration of gastric cancer cells *in vitro* and *in vivo* **A.**, The cell proliferation of MGC (i) or MKN (iii) transfected with over-expression (mGSN) or knockdown (shGSN) of gelsolin. Wound healing assay carried out with MGC (ii) or MKN (iv) with gelsolin overexpression. Representative images of triplicate experiments were shown. **B.**, Transwell assay carried out with MGC (i) or MKN (ii) with over-expression (mGSN) or knockdown (shGSN) of gelsolin. Three independent experiments with 3 fields for each were performed, and the representative fields were shown (*, *P*<0.05, student's *t*-test). **C.**, Cell adhesion assay was performed in MGC (i, ii) or MKN cell (iii) transfected with shCtrl or shGSN after 15, 30, and 60 minutes. Bar graph represents mean ±s.d. of quantification of adherent cell numbers at indicated time point (n=3; *,*P*<0.05, student's *t*-test). **D.**, Tumor size measurement of mice transplanted with GSN-overexpressed MGC cells (mGSN or control) (i), or GSN-knockdown MGC cells (shGSN or control)(ii) (n=10 per group). Results are expressed as mean ± SEM. After 13 weeks, mice were euthanized and the numbers of metastases in the lungs were counted (iii and iv).

To determine the function of gelsolin on gastric cancer cells, we established stable gastric cancer cell lines with over expression and knock down gelsolin constructs. Successful over expression or knock down of gelsolin were confirmed by western blot (Supplementary Figure S2). We found that gelsolin exerted no obvious effect on the proliferation of MGC(Figure 2A-i) and MKN (Figure 2A-iii). However, over expression of gelsolin significantly suppressed the migratory abilities of both gastric cancer cells according to wound healing assay (Figure 2A-ii, iv). Consistently, the real time cell growth and confluence curve showed that knock-down gelsolin increased the motility of MGC cell with no effect on the proliferation (Supplementary Figure S3, S4). Consistent with results obtained from wound-healing assay, over expression of gelsolin inhibited the migration of gastric cancer cells, whereas knockdown of gelsolin significantly increased migration of MGC and MKN cells by using Transwell migration assay (Figure 2B-i, ii). Highly metastatic cancer cells are believed to have enhanced adhesion ability that facilitates the migration of the cells to a new site to establish new tumors [18]. Cell adhesion assay also confirmed the inhibitory function of gelsolin on migration of gastric cancer cells (Figure 2C-i, ii, iii).

To further explore the function of gelsolin *in vivo*, MGC and MKN cell with over-expression or knockdown of gelsolin were transplanted into nude mice to observe the tumor growth and lung metastasis. We didn't find the obvious spontaneous lung metastasis in xenograft mice transplanted with MKN cells. There were no significantly difference of tumor size in MGC cell xenograft mouse model with over-expression or knockdown of gelsolin (Figure 2D-i, ii). However, the numbers of metastatic lung nodules were significantly decreased in the gelsolin-overexpression group than that in control group (Figure 2D-iii). Knockdown of gelsolin increased the lung metastasis in MGC xenograft mice (Figure 2D-iv). Taken together, our results suggest that gelsolin inhibit gastric cancer metastasis.

### Gelsolin suppress epithelial-to-mesenchymal transition of gastric cancer cells

**Figure 3:**
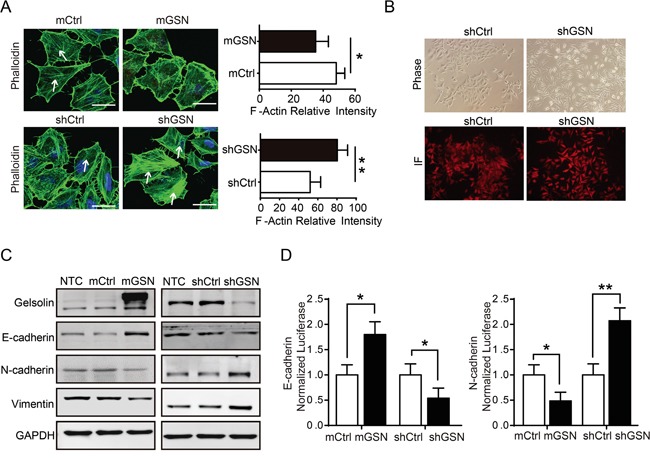
Gelsolin inhibited EMT and actin cytoskeletal rearrangement to suppress the migration of gastric cancer cell **A.**, Immunofluorescence staining for phalloidin (F-actin) in MGC cell with overexpression (mGSN) or knockdown (shGSN) of gelsolin. Scar bar, 10μm. White arrows showed the filamentous F-actin. Quantitative results of the actin fluorescence intensity were presented as mean ± SEM of triplicate experiments (*, *P*<0.05; **, *P*<0.01). **B.**, Morphology of MGC cell stably transfected with shCtrl or shGSN was examined by phase contrast microscopy and immunofluorescence (RFP). **C.**, Immunoblotting analyses of EMT-related proteins in MGC cell with overexpression or knockdown gelsolin. **D.**, Luciferase activity assay of E-cadherin or N-cadherin promoter-driven luciferase expression in indicated cells after 48h post-transfection (*, *P*<0.05; **, *P*<0.01).

Previous data have demonstrated that gelsolin mediated the remodeling of the actin cytoskeleton to suppress the metastasis of bladder cancer [12]. In gastric cancer cell, our data also showed that gelsolin modulated actin cytoskeleton remolding in MGC cell (Figure 3A). Moreover, we found a clear morphologic transition to an enhanced mesenchymal or diffuse-like pattern in MGC cell with knockdown of gelsolin(Figure 3B), which accompanied by decreased E-cadherin and increased N-cadherin and vimentin (Figure 3C, Supplementary Figure S5). The changes in cell phenotype between the epithelial and mesenchymal states, which defined as the epithelial–mesenchymal transition (EMT), are recognized as critical events for metastasis [4, 19]. To verify whether gelsolin affects EMT at the transcriptional level, luciferase assay showed that gelsolin inhibited the activity of the transfected E-cadherin promoter-reporter and enhanced the activity of the transfected N-cadherin promoter-reporter (Figure 3D).

### Inhibition of EMT by gelsolin is dependent on the p38 MAPK signaling

**Figure 4:**
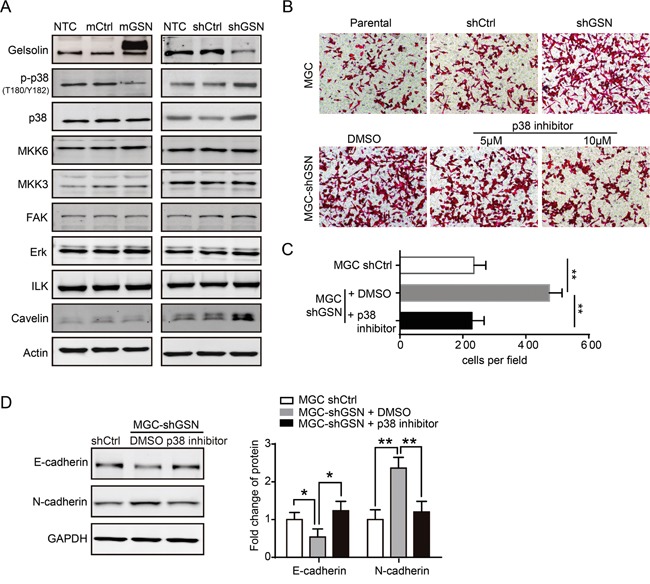
Gelsolin repressed EMT by inhibiting p38 pathway **A.**, Immunoblotting analysis of migration-related proteins in MGC cell with over-expression or knockdown of gelsolin. **B.**, Transwell assay carried out with indicated MGC cells with or without the treatment of described concentration of p38 inhibitor. **C.**, Statistical analyses of migratory cells in indicated MGC cells (**,*P*<0.01). **D.**, Immunoblotting analyses of EMT markers (E-cadherin, N-cadherin) in indicated MGC cells with control (DMSO) or p38 inhibitor (10 μM). Bar graph represents mean±s.d. of western blot quantification of E-cadherin and N-cadherin expression (n=3; *,*P*<0.05; **,*P*<0.01, student's *t*-test).

To dissect the mechanism of gelsolin on the metastasis of gastric cancer cells via EMT, we detected a series of metastasis-related proteins that were previously reported to be involved in the metastasis of gastric cancer. Western blot analysis revealed that p38 and phosphorylated p38 (p-p38) were negatively correlated with gelsolin expression in gastric cancer cell (Figure 4A). To determine the effect of p38 signaling on gelsolin-induced migration, we conducted migration assay with chemical inhibitors against p38 signaling pathway (SB203580). p38 inhibitor can reverse the inhibitory ability of gelsolin on the metastasis of gastric cancer cell (Figure 4B, 4C). With treatment of p38 inhibitor, stable knockdown of gelsolin in MGC cells increased E-cadherin levels, whereas it decreased E-cadherin levels (Figure 4D). These results suggest that p38 MAPK may be a downstream target of gelsolin in the process of EMT.

### Gelsolin inhibit PKR-p38 signaling involved in gastric cancer cell migration via interaction with PKR

**Figure 5:**
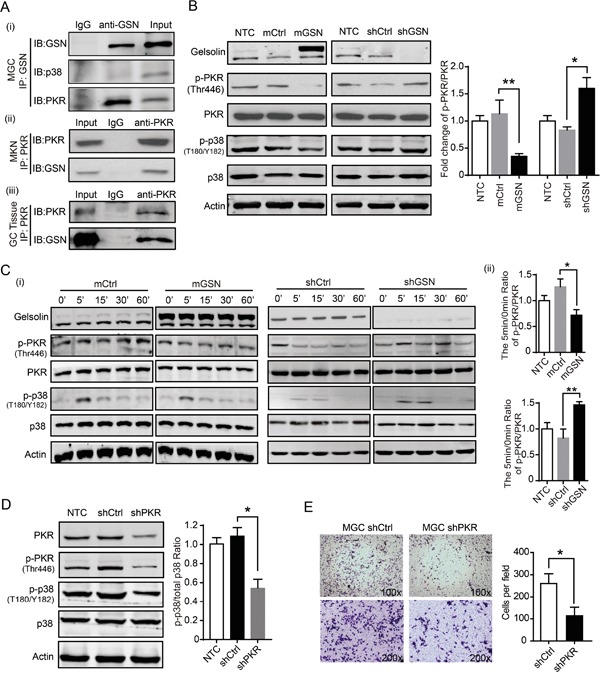
Gelsolin interacted with PKR to inhibitp38 signaling pathway **A.**, (i) Immunoprecipitation (IP) of gelsolin and immunoblotting (IB) analysis of gelsolin, PKR and p38 in MGC cell. MGC cell lysates were run as an input control, and isotype control IgG1 of gelsolin were run in the left lane. Total cell lysates were prepared from MKN cells (ii) or gastric cancer tissue from one patient with TNM stage-I gastric cancer (iii). Following immunoprecipitation with the anti-PKR antibody or IgG control antibody, total lysates and bound proteins were subjected to immunoblotting with anti-gelsolin antibody. **B.**, Western blot analyses of gelsolin, p-PKR, PKR, p-p38, and p38 proteins in indicated MGC cells. Quantification of fold change of p-PKR/PKR ratio was shown (n=3; *, *P*<0.05, student's *t*-test). **C.**, (i) Western blot analyses of p-PKR/total PKR and p-p38/total p38 from indicated MGC cells. After overnight serum starvation, the indicated MGC cells were treated with10% FBS for indicated time points and then lysed for analysis by Western blot. Representative Western blot images are shown.(ii) Bar graph represents the fold change of p-PKR/PKR at 5min time point quantified and normalized against 0 min in indicated MGC cells. (n=3; *, *P*<0.05; **, *P*<0.01, student's *t*-test). **D.**, Western blot analyses of p-p38/total p38 in MGC cells with shCtrl or shPKR for 48h. The fold change of p-p38/p38 in MGC cells with shCtrl or shPKR were quantified and normalized against untreated control cell. Values are means ± standard error means of three independent samples (*, *P*<0.05, student's *t*-test). **E.,** Transwell assay carried out with MGC cell transfected knockdown of PKR. Representative Transwell images and statistical data were shown (n=3; *, *P*<0.05, student's *t*-test).

To determine the molecular mechanism of gelsolin on the p38 signaling pathway, an immunoprecipitation (IP) assay was performed. We fund that there was no direct interaction of gelsolin and p38 protein in MGC cell (Figure 5A-i). To identify proteins that interact with gelsolin, we identified interacted proteins by mass spectrometry. The dsRNA-dependent kinase PKR was identified in this procedure. PKR has been shown to interact with gelsolin during the innate immune response [20], so we tested whether gelsolin interacted with PKR in gastric cancer cells. IP data identified that gelsolin directly bound to PKR in MGC (Figure 5A-i) and MKN cells (Figure 5A-ii). To support this hypothesis, IP assay also showed the interaction of gelsolin and PKR in clinical patient tissues with higher gelsolin expression (Figure 5A-iii). These results suggest that PKR may be a target of gelsolin. Furthermore, we determined the expression of phospho-PKR/PKR and phospho-p38/p38 in stable transfected MGC-mGSN and MGC-shGSN cells and control cells. Our results showed that the knockdown of gelsolin expression triggered a higher effect on the expression of endogenous phosphorylated PKR in MGC cell (Figure 5B). Serum stimulation decreased the activation of PKR in MGC-mGSN stable cell and increased the activation of PKR in MGC cell with knockdown of GSN (Figure 5C-i, ii).

PKR has been reported to be closely associated with tumor progression [21–23]. However, the effects of PKR on gastric cancer cells have not been characterized. There was no significant effect of PKR on the proliferation of MGC cell (Supplementary Figure S6). Knockdown of PKR significantly decreased the expression of phospho-p38 and p38 in MGC cell (Figure 5D). Knockdown PKR and inactivation of phospho-PKR was accompanied by reducing the migration of MGC cell (Figure 5E). Taken together, these results suggest that the interaction of gelsolin with PKR inhibit the PKR-p38 signaling to suppress the migration of gastric cancer cell.

### Downregulated gelsolin was associated with increased PKR-p38 signaling proteins in human gastric cancer

**Figure 6:**
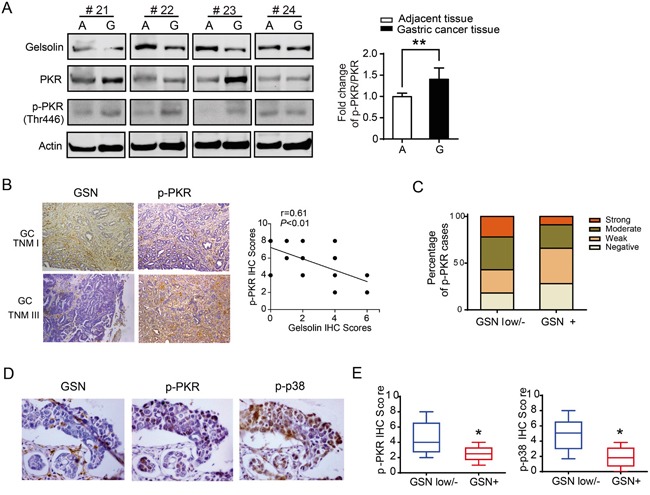
Downregulated gelsolin was associated with increased expression of PKR-p38 signaling proteins in human gastric cancer tissues **A.**, Representative Western blot images of gelsolin, PKR and pPKR protein levels in 4 cases of human gastric cancer tissue (G) and matched adjacent tissue (A) were shown. Bar graph represents mean ±s.d. of western blot quantification of p-PKR/PKR in gastric cancer tissues and matched adjacent tissues (n=20; *, *P*<0.05, student's *t*-test). **B.**, Representative IHC image of gelsolin and p-PKR from TNM I case and TNM III case were shown (100x), and the correlation between gelsolin and p-PKR in 20 cases of gastric cancer tissues was analyzed. **C.**, The percentage of p-PKR expression was shown in gastric cancer patients with lower/negative or positive gelsolin expression(*P*<0.05; *x^2^* test). **D.**, Representative IHC images of gelsolin, p-PKR, and p-p38 from consecutive gastric cancer tissue sections were shown (400x). **E.**, the box and whisker plots showed IHC scores of p-PKR and p-p38 with various gelsolin expression intensities (*, *P*<0.05).

To verify the relationship of gelsolin and PKR-p38 signaling pathway in human gastric cancer tissues, we detected the expression of p-PKR and PKR protein in gastric cancer and their corresponding non-tumorous tissues by Western blot. Our results showed that p-PKR and PKR were significantly upregulated in gastric cancer (*P*<0.01; Figure 6A). Moreover, immunohistochemical staining data demonstrated that gastric cancer with lower gelsolin exhibited higher expression of p-PKR (Figure 6B, 6C). The correlation assay showed that loss expression of gelsolin was associated with increased phosphorylated PKR and p38 expression in human gastric cancer tissues (Figure 6D, 6E; Supplementary Table S1).

## DISCUSSION

**Figure 7:**
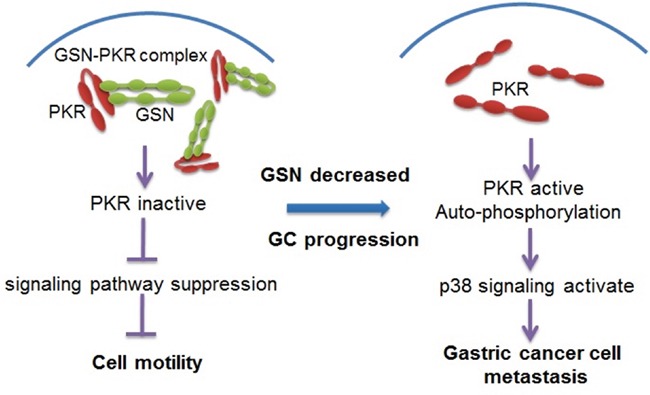
Proposed model for the mechanism of gelsolin signaling pathway in gastric cancer cell metastasis In non-metastatic gastric cancer cells, gelsolin interacts with PKR and inhibits the activation of PKR. However, in metastatic cancer cells, gelsolin is down-regulated, which allows for the autophosphorylation of PKR. PKR activation leads to the activation of p38 protein and downstream signaling pathway to promote the metastasis of gastric cancer.

Here, our data show that gelsolin plays a previously unrecognized role in cancer metastasis through inhibition of the PKR-p38 signaling pathway. On the basis of the above experimental and clinical results, we hypothesize that gelsolin normally interacts with PKR and inhibit the phosphorylation of PKR to inhibit metastasis-related signaling. However, in metastatic gastric cancer, down-regulated gelsolin releases PKR to activate p38 signaling pathway to promote the metastasis of gastric cancer (Figure 7).

Gelsolin evidently plays vital roles in many different pathological processes [9, 20, 24–26]. Numerous published reports have shown that silencing gelsolin might play a role in cancer development [10]. Gelsolin is a cytoskeleton-associated protein that regulates actin [27, 28], and participates in the coordinated regulation of several signal transduction pathways [6]. Our finding indicates a role for gelsolin in the repression of gastric cancer metastasis. EMT transition is a critical component of cancer progression that facilitates the development of metastasis [29]. Gelsolin is a critical mediator of p38-dependent EMT. The p38 MAPK signaling pathway plays important roles in the ability of cells to integrate external cues and to respond appropriately [30, 31]. We previously have shown that the p38-HSP27 signaling pathway is involved in tumor metastasis [32]. In this study, we illustrate in detail that the p38 signaling pathway can be regulated by gelsolin to promote the process of EMT, which in turn leads to the metastasis of gastric cancer.

New insights into gelsolin-mediated signal transduction have been discovered. Therefore, our detailed research identified PKR, a dsRNA-activated protein kinase, as the binding protein of gelsolin that can regulate the p38 signaling pathway. PKR was originally identified as a component of the interferon-induced antiviral response [33], and involved in the regulation of cellular and viral protein synthesis [34]. PKR activation blocks global protein synthesis, leading to cell death inresponse to a variety of cellular stresses. We did not observe the significant effect of PKR on the proliferation of gastric cancer cells. This is may be due to the dual role of PKR on activating the nuclear factor-kB pathway to promote cell proliferation [35]. The activation of PKR requires that it undergo dimerization and intermolecular auto-phosphorylation [36]. PKR is also critical to regulate the inhibition/activation of p38 MAPK [37]. As for the function of PKR in cancer cells, the results are still controversial [38]. In this study, our data indicated that gelsolin mediated the suppression of metastasis that can be attributed at least in part to the inhibition of PKR-p38 signaling in gastric cancer.

In summary, this evidence suggests that the dysregulation of the p38 signaling pathway by gelsolin is an important mechanism that underlies gastric cancer metastasis. In clinical gastric cancer tissue samples, the upregulated expression levels of PKR-p38 signaling pathway proteins were associated with low levels of gelsolin, which correlated with tumor metastasis status. Of course, we need to note that confirmation of these findings in a large prospective cohort of patients who are uniformly staged and treated is desirable. These signaling proteins may serve as potential treatment targets for the modulation of this pathway in metastatic disease.

## MATERIALS AND METHODS

### Patients and tissue samples

In all, 56 gastric tumors were obtained from a cohort of patients who were treated at Xinhua Hospital, Shanghai Jiao Tong University School of Medicine, China, between 2006 and 2009. Fresh gastric cancer tissues and matched adjacent tissues from 20 post operational patients with gastric cancer were also collected and immediately snap-frozen for protein analyses or pathological progress. All patients were diagnosed by pathological analyses based on the International Union against Cancer (UICC) defined TNM criteria. The study protocol conformed to the ethical guidelines of the Declaration of Helsinki and was approved by the Institutional Review Board and Ethics Committee of Xinhua Hospital. Before inclusion in the study, all patients provided written informed consent.

### Cell culture and treatment conditions

The human gastric cancer cell lines MGC-803 (MGC) were obtained from the Chinese Academy of Sciences Cell Bank of Type Culture Collection. The human gastric cancer cell line MKN-45 (MKN) was provided by the Beijing Institute for Cancer Research. All cell lines were routinely tested and authenticated in November, 2013, using a panel of genetic and epigenetic markers. The cells used in our experiments were in the log phase of growth and were negative for Mycoplasma and endotoxin, as confirmed by PCR with the Mycoplasma Tissue Culture Detection kit (Gen-Probe) and the Limulus Amebocyte Lysate assay (Cambrex), respectively. The cells were routinely cultured in DMEM media supplemented with 10% fetal calf serum, 100 U/ml penicillin and 100 μg/ml streptomycin (Gibco). Cells were maintained at 37 °C, 5% CO_2_ in a humidified incubator. In some experiments, gastric cancer cells were treated with trichostatin A (TSA) or 5-aza-2-deoxycytidine (5-Aza-dc) for western blot.

### *In vivo* tumor growth and metastasis assays

To examine the tumor growth and metastatic ability of the gastric cancer cell overexpressing or knocking down gelsolin in animals, 1.0 x 10^6^ indicated gastric cancer cell (MGC) in 0.1 mL of PBS were inoculated subcutaneously into nude mice. Tumor growth was routinely monitored using a common caliper for the following 13 weeks. Tumor volume was calculated with the following formula: volume = 0.5 × width^2^ × length. The mice were sacrificed at the end of observation, and metastatic nodal in lung of dedicated mice were histologically detected and counted. All experiments contained 10 mice per group. The animal protocol was approved by the Institutional Committee of Shanghai Jiao Tong University School of Medicine for Animal Research.

### Immunohistochemical staining

Standard immunohistochemical procedures were performed for the expression of gelsolin with the VECTASTAIN Elite ABC system (Vector Laboratories, USA) according to the manufacturer's protocol. All protocols have been described previously [12]. An anti-gelsolin monoclonal antibody (Sigma, USA) was used as the primary antibody. The staining intensity and the proportion of stained cells were semi-quantitatively determined. The intensity and the percentage of positive cells were multiplied to obtain a score (0-12) as previously described [12]. All slides were scored by two observers who were blinded to the pathology and the clinical features. In cases in which the score difference was greater than or equal to 2, the slides were re-examined until a consensus was reached by the observers.

### Western blot analysis and immunoprecipitation

Western blot was performed as previously described [12, 32]. Briefly, the cells were lysed in equal volumes of ice cold lysis buffer and a protease inhibitor cocktail. Cell lyses were separated by SDS-PAGE. After overnight incubation at 4°C with anti-PKR, anti-phospho-PKR (Thr446), anti-p38, anti-phospho-p38 MAPK (Thr180/Tyr182) or other indicated antibodies (Cell Signaling Technology), the membranes were incubated with IRDye 800 or IRDye 680 secondary antibodies (LI-COR Biosciences, USA). For immunoprecipitation, total cell lysates were incubated with anti-gelsolin, anti-PKR or isotype IgG1, and proteins were pulled down using agarose beads. Protein samples were immunoblotted with the indicated antibodies. The targeted proteins were detected and quantified on a Li-COR Odyssey infrared imaging system (LI-COR Biosciences).

### Cell proliferation

Proliferation was performed as previously described [39]. Briefly, gastric cancer cells were plated and maintained overnight in 96-well plates at a density of 3×10^3^ cells per well and were incubated with fresh medium. Cell viability was determined with the CCK-8 Cell Proliferation Assay (Dojindo, Japan) according to the manufacturer's instructions. Moreover, in order to verify the rate of gastric cancer cell proliferation, in several cases *in vitro* real time microscopy observation was performed using JuLi Live cell analyzer (NanoEnTek, Korean). The images were performed every 10 min for 96 hours depending on a case.

### Protein expression constructs and luciferase assay

Plasmid mcherry-GSN (mGSN), shGSN, and respective control constructs were purchased from OriGene Technologies (USA). Plasmid mcherry-PKR (mPKR), shPKR, and respective control vectors were provided by Shanghai GeneChem Co., Ltd. (Shanghai, China). E-Cadherin and N-cadherin promoter activity was determined using E-cadherin or N-cadherin luciferase reporter construct, which encoding the human E-cadherin 5' promoter (−178 to +92) or N-cadherin 5' promoter (−860 to +20) in pGL3basic (Promega, Madison, WI, USA). The pRL-TKvector was used as an internal control. Cancer cells (80–90% confluent) were transfected with respective vectors according to the manufacturer's instructions.

### Migration assay and cell adhesion assay

Wound healing assay and migration assay was performed as previously described [12]. Leica microscope was used to capture images. The number of cells that had passed through the membrane was counted using Image J software (NIH, Rockville, MD, USA). The transfected cell adhesion to Matrigel were evaluated by counting. MGC cells transfected with shCtrl or shGSN were trypsinized, washed twice in 1× PBS, and then 1×10^4^ cells with100 μl medium were plated into every well in a 96-well plate pre-coated with 40 μl of 50 μg/ml Matrigel and the cells were incubated at 37°C for indicated times. The non-adherent cells were washed out 3 times with PBS, and the adherent cells were fixed to stain by crystal violet solution. The number of cells that adhere to Matrigel were counted under an inverted microscope (Olympus, Japan) at 100×magnification from 3 randomly selected fields in each well. The independent experiments were run in three times.

### Fluorescence microscopy

For immunofluorescence staining, gastric cancer cells were cultured at up to 50-60% confluence before being fixed and permeabilized. Cellular F-actin was visualized by staining with Alexa 488 phalloidin (LifeTechnologies, USA) according to the manufacturer'sguidelines. Cells were mounted with ProLong® Gold antifade Reagent with DAPI (LifeTechnologies, USA). Images were captured using Leica SP5 Laser scanning confocal microscope.

### Statistical analysis

Statistical significance between groups was determined by a two-tailed Student's t test and a two-way ANOVA test. Differences were considered to be significant when *P*<0.05. For the survival analysis, Kaplan-Meier survival curves were generated, and all statistical data were analyzed for statistical significance with GraphPad Prism 5.0 for Windows (GraphPad Software, USA).

## SUPPLEMENTARY MATERIALS FIGURES AND TABLE


