# Supplementary material for: Gelsolin suppresses gastric cancer metastasis through inhibition of PKR-p38 signaling
Source: Oncotarget. 2016 Jul 13;7(33):53459–70. doi: 10.18632/oncotarget.10557 (PMC5288199; doi:10.18632/oncotarget.10557)
Supplement: Supplementary file 1 [file oncotarget-07-53459-s001.pdf]

Gelsolin suppresses gastric cancer metastasis through inhibition of PKR-p38 signaling

SUPPLEMENTARY FIGURES AND TABLE

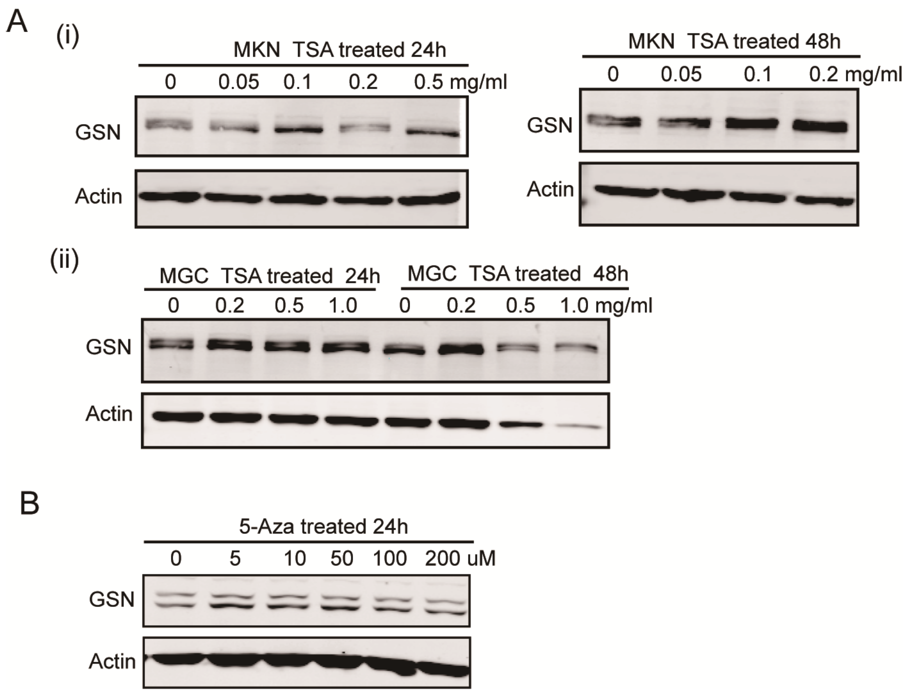

Supplementary Figure S1: DNA methylation and histone deacetylation account for the down regulation of GSN in gastric cancer cells. A., GSN expression can be reconstituted in MKN-45 (i) and MGC-803 (ii)gastric cancer cells by treatment with the histone deacetylase inhibitor, Trichostatin A (TSA). **B.** WB test showed that the DNA methylation inhibitor, 5-aza-2'-deoxycytidine (5-Aza), can increase the GSN expression in MGC-803 gastric cancer cell. To ensure equal loading, the blots were stripped and immunoblotted with an antibody against actin.

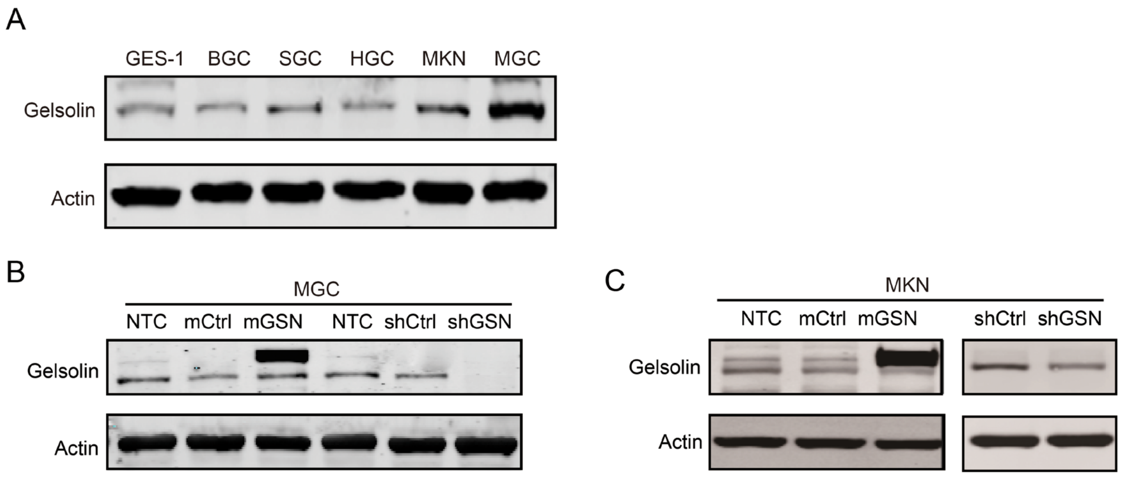

Supplementary Figure S2: Immunoblotting analysis of gelsolin expression in gastric cancer cell lines (A). The efficiency of over expression or knockdown gelsolin in MGC (B) and MKN cell (C).

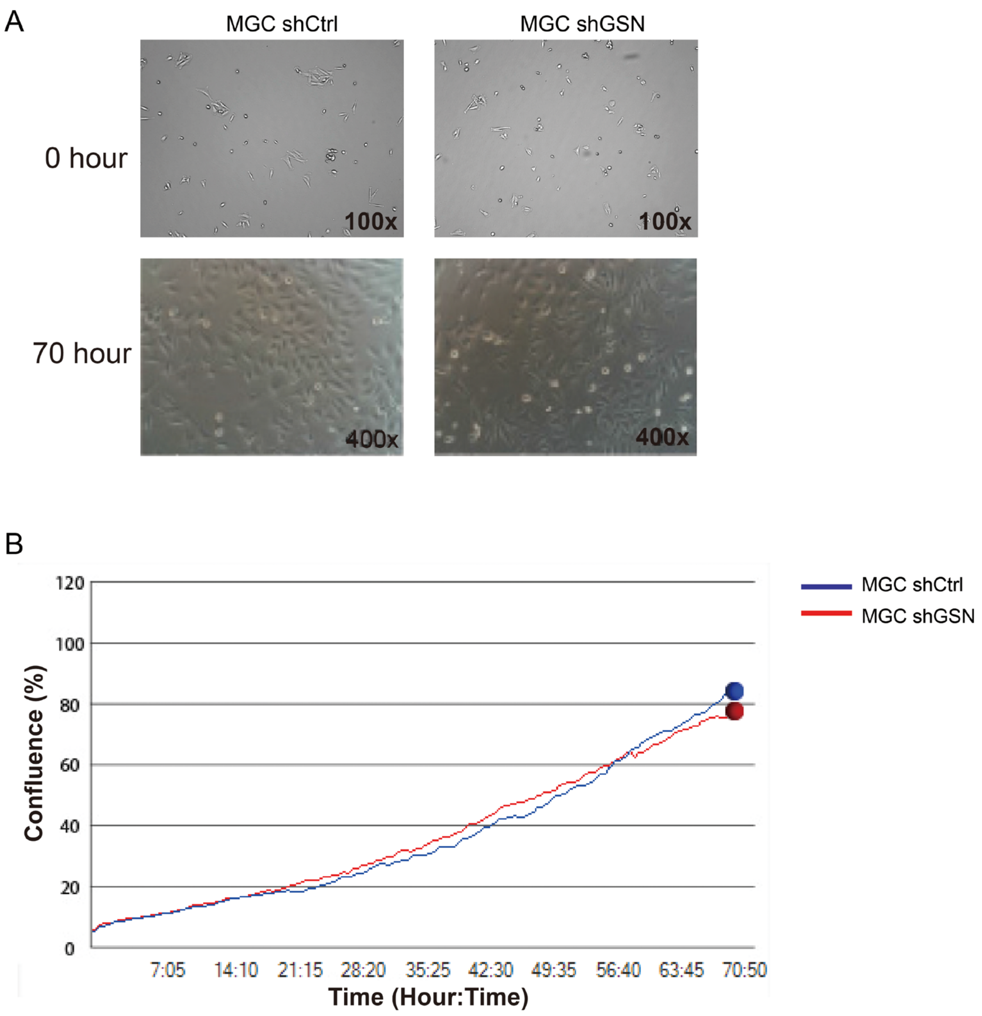

Supplementary Figure S3: Knockdown of GSN have no effect on the growth of MGC gastric cancer cell. A., MGC cells were transfected with scramble (shCtrl) or shGSN respectively, then cultured in 6-well plate in same density. Represent phase images showed the cell density on the time of 0h and 70h. **B.**, The real-time cell proliferation of MGC-shCtrl and MGC-shGSN was observed and the cell numbers were counted by JuLi Live cell analyzer (NanoEnTek). The cell proliferation curve showed there were no significant difference in the growth of MGC-shCtrl and MGC-shGSN.

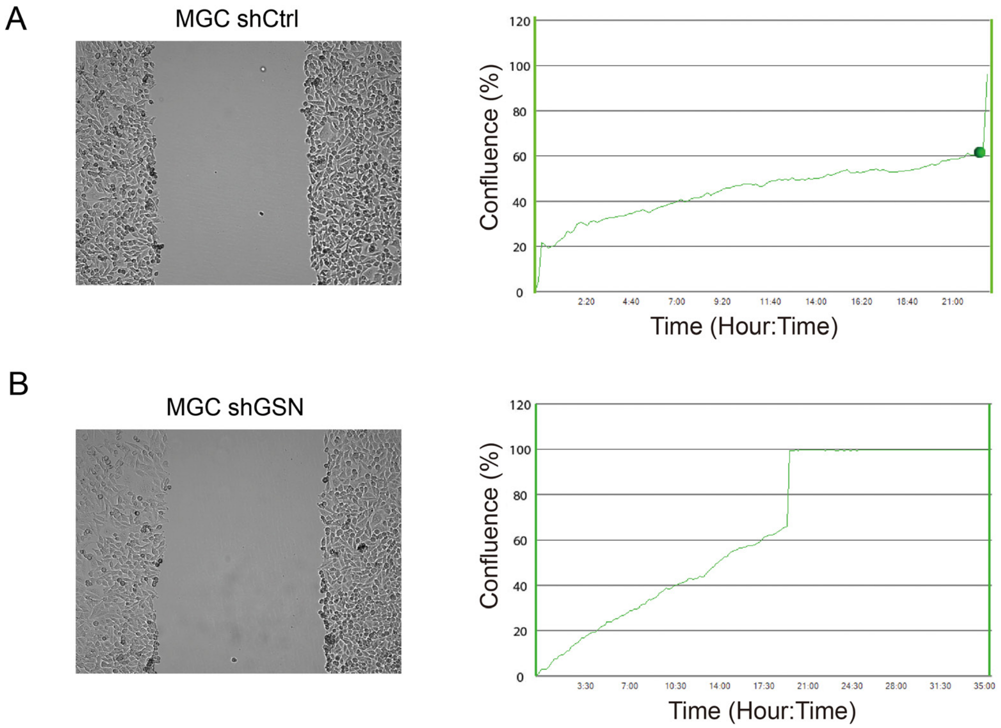

Supplementary Figure S4: Knockdown of GSN increased the motility of MGC cell. MGC cell were transfected with scramble (shCtrl) **A.** or shGSN **B.** respectively, then cultured in 24-well plate in same density. When the cell density were at 100% confluence, scratch assay were used to analyze the rate of wound healing. Represent phase (Left image) show the cell density on the time point of 0h. The real-time cell wound closure of MGC-shCtrl (A, right) and MGC-shGSN (B, right) cells were observed over 36 h by JuLi Live cell analyzer (NanoEnTek). The confluent curves showed that MGC-shGSN cell have faster wound closure ability than MGC-shCtrl cell.

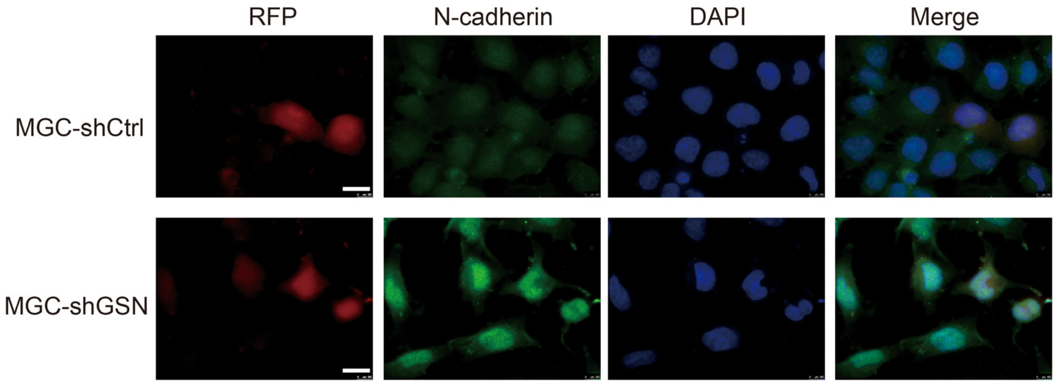

Supplementary Figure S5: Immunofluorescence staining of N-cadherin in MGC cell with shCtrl or shGSN. Red: stable MGC cells with shCtrl or shGSN; Green: N-cadherin; Blue: DAPI.

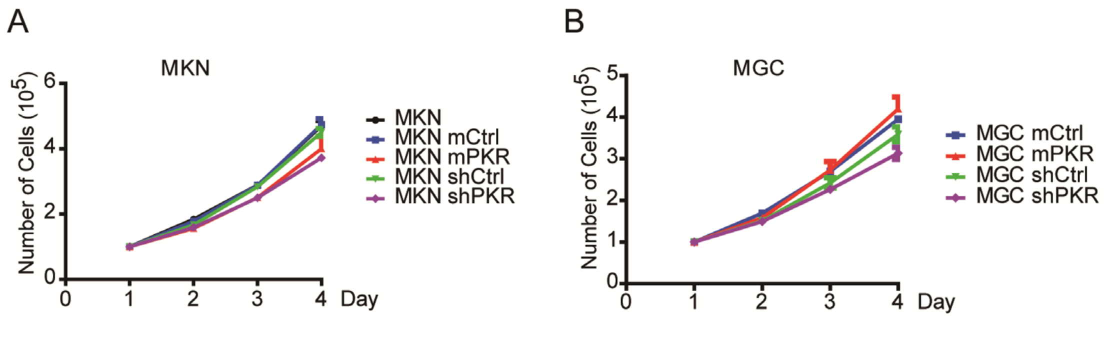

Supplementary Figure S6: Effect of PKR on the proliferation of MGC-803 and MKN-45 gastric cancer cell lines. Cell proliferation was determined by cell counting. There was no significant difference in the numbers of MKN-45 **A.** and MGC-803 cell **B.** with over expression or knock-down of PKR.

Supplementary Table S1: Relationship between GSN and phospho-PKR, p38 expression in gastric cancer

	GSN expression			*P* value
	All	Positive	Negative	
p-PKR expression				
High	41	19 (46%)	22 (54%)	
Low	15	13 (87%)	2 (13%)	0.01
p38 expression				
High	38	18 (47%)	20 (53%)	
Low	18	14 (78%)	4 (22%)	0.04
Metastasis status				
Negative	44	29 (66%)	15 (34%)	
Positive	12	3 (25%)	9 (75%)	0.02
